# Synergistic and Additive Effect of Oregano Essential Oil and Biological Silver Nanoparticles against Multidrug-Resistant Bacterial Strains

**DOI:** 10.3389/fmicb.2016.00760

**Published:** 2016-05-23

**Authors:** Sara Scandorieiro, Larissa C. de Camargo, Cesar A. C. Lancheros, Sueli F. Yamada-Ogatta, Celso V. Nakamura, Admilton G. de Oliveira, Célia G. T. J. Andrade, Nelson Duran, Gerson Nakazato, Renata K. T. Kobayashi

**Affiliations:** ^1^Laboratory of Basic and Applied Bacteriology, Department of Microbiology, Center of Biological Sciences, Universidade Estadual de LondrinaLondrina, Brazil; ^2^Laboratory of Molecular Biology of Microorganisms, Department of Microbiology, Center of Biological Sciences, Universidade Estadual de LondrinaLondrina, Brazil; ^3^Laboratory of Technological Innovation in Drug and Cosmetics Development, Department of Basic Health Sciences, Center of Health Sciences, Universidade Estadual de MaringáMaringá, Brazil; ^4^Laboratory of Microbial Ecology, Department of Microbiology, Center of Biological Sciences, Universidade Estadual de LondrinaLondrina, Brazil; ^5^Laboratory of Electron Microscopy and Microanalysis, Department of General Biology, Center of Biological Sciences, Universidade Estadual de LondrinaLondrina, Brazil; ^6^Institute of Chemistry, Universidade Estadual de CampinasCampinas, Brazil

**Keywords:** oregano oil, biological silver nanoparticles, multidrug-resistant bacteria, synergism, antibacterial

## Abstract

Bacterial resistance to conventional antibiotics has become a clinical and public health problem, making therapeutic decisions more challenging. Plant compounds and nanodrugs have been proposed as potential antimicrobial alternatives. Studies have shown that oregano (Origanum vulgare) essential oil (OEO) and silver nanoparticles have potent antibacterial activity, also against multidrug-resistant strains; however, the strong organoleptic characteristics of OEO and the development of resistance to these metal nanoparticles can limit their use. This study evaluated the antibacterial effect of a two-drug combination of biologically synthesized silver nanoparticles (bio-AgNP), produced by Fusarium oxysporum, and OEO against Gram-positive and Gram-negative bacteria, including multidrug-resistant strains. OEO and bio-AgNP showed bactericidal effects against all 17 strains tested, with minimal inhibitory concentrations (MIC) ranging from 0.298 to 1.193 mg/mL and 62.5 to 250 μM, respectively. Time-kill curves indicated that OEO acted rapidly (within 10 min), while the metallic nanoparticles took 4 h to kill Gram-negative bacteria and 24 h to kill Gram-positive bacteria. The combination of the two compounds resulted in a synergistic or additive effect, reducing their MIC values and reducing the time of action compared to bio-AgNP used alone, i.e., 20 min for Gram-negative bacteria and 7 h for Gram-positive bacteria. Scanning electron microscopy (SEM) revealed similar morphological alterations in Staphylococcus aureus (non-methicillin-resistant S. aureus, non-MRSA) cells exposed to three different treatments (OEO, bio-AgNP and combination of the two), which appeared cell surface blebbing. Individual and combined treatments showed reduction in cell density and decrease in exopolysaccharide matrix compared to untreated bacterial cells. It indicated that this composition have an antimicrobial activity against S. aureus by disrupting cells. Both compounds showed very low hemolytic activity, especially at MIC levels. This study describes for the first time the synergistic and additive interaction between OEO and bio-AgNP produced by F. oxysporum against multidrug-resistant bacteria, such as MRSA, and β-lactamase- and carbapenemase-producing Escherichia coli and Acinetobacter baumannii strains. These results indicated that this combination can be an alternative in the control of infections with few or no treatment options.

## Introduction

Bacterial antimicrobial resistance to most conventional antibiotics has become a clinical and public health problem. Infections due to multidrug-resistant microorganisms, such as bacteria that produce extended-spectrum β-lactamases (ESBL) and carbapenemases (*Klebsiella pneumoniae* carbapenemase, KPC) and MRSA can be challenging to control leading to high treatment costs, therapeutic failure and death (Silva and Lincopan, 2012; Cantas et al., 2013).

ESBL and KPC hydrolyze the β-lactam ring resulting in an inactive antimicrobial (Queenan and Bush, 2007; Drawz and Bonomo, 2010). ESBLs mediate resistance to most β-lactams, mainly in Gram-negative bacteria (Dhillon and Clark, 2012; Silva and Lincopan, 2012). In these cases, carbapenem antibiotics, such as imipenem, meropenem, and ertapenem, are drugs of choice for treatment. However, carbapenemases reduce treatment options because they inactivate penicillins, cephalosporins, monobactams, and carbapenems (Queenan and Bush, 2007).

Usually ESBLs and KPC are encoded by genes carried by mobile genetic elements which also carry resistance genes to other antimicrobial agents, contributing to the emergence of multidrug resistance and its rapid spread between different strains and species (Pitout, 2012; Silva and Lincopan, 2012; Patel and Bonomo, 2013; Shaikh et al., 2015). ESBL- and KPC-producing strains usually exhibit resistance to quinolones, tetracyclines, cotrimoxazol, trimethoprim, and aminoglycosides (Dhillon and Clark, 2012; Pitout, 2012; Patel and Bonomo, 2013).

In Gram-positive bacteria, the most common mechanism of resistance to β-lactam occurs due a mutant transpeptidase gene. Methicillin resistance in *S. aureus* occurs because of the *mec*A gene, which encodes transpeptidases that have low affinity for β-lactam antibiotics (Rice, 2012). MRSA strains can harbor resistance genes for other antimicrobials besides *mec*A, reducing treatment options (Turlej et al., 2011).

Multiresistant bacteria have been found in foods for human consumption, streams and effluents and thus it is a hospital, community, and environmental problem (Prado et al., 2007; Fontes et al., 2011; Silva and Lincopan, 2012; Rubin et al., 2014; Koga et al., 2015). As soon as new β-lactams are widely used, new β-lactamases are also discovered (Bush, 2010; Kong et al., 2010; Shaikh et al., 2015). Therefore, there is crucial need for research and development of new drugs with potential to combat resistant strains to minimize their selection.

Compounds from natural sources such as animals, plants, and microorganisms have been proposed as potential antimicrobial alternatives (Mandal and Mandal, 2011; Cardozo et al., 2013; Biasi-Garbin et al., 2015). Spice essential oils have been documented as being antimicrobial (Burt, 2004; Du et al., 2009; Betancourt et al., 2012; Nazzaro et al., 2013). Oil phenolic substances are mainly responsible for antibiotic properties (Rhayour et al., 2003; Burt, 2004; Nostro et al., 2007; Hyldgaard et al., 2012).

Studies on the antibacterial mechanisms of action of plant essential oils, including the OEO, suggest that hydrophobic bioactive compounds cause damage to cell membrane, increase cell permeability, affect ATP production, act on protein synthesis, cause cellular pH disturbance, induce cytoplasmic changes and interfere with quorum sensing (Helander et al., 1998; Ultee et al., 1999; Lambert et al., 2001; Rhayour et al., 2003; Souza et al., 2010, 2013; Szabó et al., 2010; Hyldgaard et al., 2012).

OEO, extracted mainly from the leaves of the herb *O. vulgare* by ecofriendly methods, has been reported as having broad antibacterial activity (Burt, 2004; Busatta et al., 2007; Betancourt et al., 2012). Other biological activities such as antifungal, antiviral, antioxidant and anticancer have been described for OEO (Cervato et al., 2000; Kalemba and Kunicka, 2003; Hyldgaard et al., 2012; Gautam et al., 2014; Gilling et al., 2014; Sobral et al., 2014).

Carvacrol and thymol are the main components of OEO (Nostro et al., 2004; Cleff et al., 2008; Hyldgaard et al., 2012; Stojković et al., 2013), and the antimicrobial activity of this oil varies according to their amounts. Synergistic and additive interactions between carvacrol and thymol have been reported (Bassolé and Juliani, 2012; Hyldgaard et al., 2012); furthermore, a mixture of compounds with antimicrobial activity could minimize the selection of resistant strains. Studies have shown that OEO has the potential to prevent food from being contaminated and control worrisome hospital infections (Nostro et al., 2004; Si et al., 2008; Barros et al., 2009; Amrouni et al., 2014; Honório et al., 2015). Despite the potential antimicrobial activity of OEO, its strong taste and smell seem to limit its use, so alternatives are needed to minimize or eliminate such undesirable characteristics (Burt, 2004; Alvarez et al., 2014).

Silver has been used for millennia to treat wounds and eye infections and to preserve food and water (Alexander, 2009). Nanotechnology has proved to be a useful tool for solving biomedical problems. Silver nanoparticles have been intensively studied as antimicrobial agents, including their use against multidrug-resistant bacteria (Li et al., 2010; Cardozo et al., 2013; Naqvi et al., 2013; Ansari et al., 2014; Bibbs et al., 2014; Palanisamy et al., 2014; Singh et al., 2014; Subashini et al., 2014; Theophel et al., 2014). Through nanotechnology, it has been possible to revive the use of silver to combat resistant bacteria. Nanoscale enhances the antibacterial activity of silver even at low concentrations; nanometer metallic particles show altered physical, chemical and biological properties compared to conventional silver, due to their high surface-to-volume ratio (Nowack et al., 2011; Rai et al., 2012; Herman and Herman, 2014). Furthermore, silver nanoparticles have been reported to be less toxic than silver ions to host (de Lima et al., 2012).

The bactericidal mechanism of silver nanoparticles is not clearly understood; however, it is believed that there is a multiple mechanism of action (Herman and Herman, 2014). Researchers have suggested that silver nanoparticles cause cell membrane disintegration and increase cell surface permeability, inactivate bacterial enzymes by interaction with thiol groups, deplete levels of intracellular ATP, cause DNA damage and induce free radical formation (Feng et al., 2000; Dibrov et al., 2002; Lok et al., 2006; Li et al., 2010; Kim et al., 2011; Theophel et al., 2014).

Although silver nanoparticles show excellent antimicrobial activity, silver-resistant bacteria have been described; and these microorganisms can quickly develop resistance to silver nanoparticles by genetic alterations (Losasso et al., 2014; Graves et al., 2015).

The combination of several antimicrobials seems to be the best strategy for controlling emergence of antibiotic-resistant microorganisms (Fischbach, 2011; Bass et al., 2015; Bollenbach, 2015). Many studies show additive or synergistic antibacterial effects of silver nanoparticles combined with alternative (eugenol, phenazine-1-carboxamide, and cinnamaldehyde) and conventional (ampicillin, kanamycin, erythromycin, chloramphenicol, amoxicillin, ciprofloxacin, and moxifloxacin; Li et al., 2005; Fayaz et al., 2010; Cardozo et al., 2013; Ghosh et al., 2013; Theophel et al., 2014; Biasi-Garbin et al., 2015) antimicrobial compounds. Synergistic and additive antimicrobial effects have been reported for OEO or its components combined with eugenol, linalool, menthol, cinnamaldehyde, *Thymus vulgaris* essential oil, *Rosmarinus officinalis* essential oil, gentamicin, polymyxin, kanamycin, levofloxacin or many others (Rosato et al., 2007; Si et al., 2008; de Souza et al., 2009; Bassolé and Juliani, 2012; Stojković et al., 2013; Honório et al., 2015).

Biological systems have been extensively used for rapid and eco-friendly biosynthesis of silver nanoparticles; instead of using chemical reagents, these processes involve fungi, bacteria, plants and other organisms or biomolecules (Sastry et al., 2003; Durán et al., 2005, 2011; Sankar et al., 2013; Singh et al., 2014). This study evaluated, for the first time, the antibacterial effect of a two-drug combination composed of bio-AgNP synthesized by *F. oxysporum* and OEO against standard and resistant Gram-positive and Gram-negative strains.

## Materials and methods

### Bacterial strains

All antimicrobial assays were performed against reference strains from American Type Culture Collection (ATCC) provided by the Laboratory of Basic and Applied Bacteriology of Londrina Stated University (Londrina, Paraná, Brazil), and clinical strains provided from Londrina University Hospital (Londrina, Paraná, Brazil). The standard bacterial strains used were as follows: methicillin-sensitive *S. aureus* (ATCC 25923), *Streptococcus mutans* (ATCC 25175), *E. coli* (ATCC 25922), *K. pneumoniae* (ATCC 10031), *K. pneumoniae* (ATCC 700603), *Salmonella enterica* serovar Enteritidis (ATCC 13076), *S. enterica* serovar Typhimurium UK-1 (ATCC 68169) and MRSA N315 provided by Dr. Elza Masae Mamizuka (São Paulo University, São Paulo – SP, Brazil). Several clinical isolates obtained from Londrina University Hospital were also tested (Table 1). Four isolates of ESBL-producing *E. coli* and two isolates of carbapenemase-producing *E. coli* from urinary tract infections (provided by Dr. Eliana Carolina Vespero, Londrina University Hospital, Londrina); one isolate of carbapenem-resistant *A. baumannii* from inguinal-rectal swabs (provided by Dr. Floristher Elaine Carrara Marroni, Londrina Clinics Hospital, Londrina) and two isolates of MRSA strains from secretions-general discharges (provided by Dr. Marcia Regina Eches Perugini, Londrina University Hospital, Londrina). The bacterial strains were stored in 25% glycerol (Merck) at −80°C.

**Table 1:** **Susceptibility to antibiotics of clinical strains**.

***S. aureus***							**PEN**	**OXA**	**CIP**	**RIF**	**GEN**	**STR**	**TET**	**ERY**	**CLI**	**LNZ**	**SXT**
MRSA 101							R	R	R	S	S	S	S	R	R	S	S
MRSA 107							R	R	R	S	R	R	S	R	R	S	R
***E. coli***	**PTZ**	**CEP**	**CFZ**	**CTX**	**CAZ**	**CPM**	**AZT**	**IPM**	**MRP**	**ERT**	**NOR**	**CIP**	**LVX**	**GEN**	**AMI**	**SXT**	**NIT**
ESBL 167	S	–	R	R	R	R	R	S	S	S	–	R	R	S	S	R	S
ESBL 169	S	R	–	R	R	R	R	S	S	S	R	R	S	S	S	S	S
ESBL 176	S	R	–	R	R	R	I	S	S	S	–	S	S	S	S	S	S
ESBL 192	S	S	–	R	R	I	S	-	S	S	–	R	R	S	S	S	S
KPC 131	R	–	–	R	S	S	R	R	R	R	–	R	–	–	S	–	–
KPC 133	I	–	–	R	I	I	R	S	R	R	–	R	–	–	S	–	–
***A. baumannii***			**PTZ**	**AMS**	**CTX**	**CAZ**	**CPM**	**AZT**	**IPM**	**MRP**	**ERT**	**CIP**	**LVX**	**GEN**	**AMI**	**TET**	**SXT**
CR 01			R	R	R	R	R	R	R	R	R	R	R	R	R	R	R

*AMI, amikacin; AMS, ampicillin + sulbactam; AZT, aztreonam; CAZ, ceftazidime; CEP, cephalothin; CFZ, cefazolin; CIP, ciprofloxacin; CLI, clindamycin; CPM, cefepime; CTX, cefotaxime; ERY, erythromycin; ETP, ertapenem; GEN, gentamicin; IPM, imipenem, LNZ, linezolid; LVX, levofloxacin; MRP, meropenem; NIT, nitrofurantoin; NOR, norfloxacin; OXA, oxacillin; PEN, penicillin; PTZ, piperacillin + tazobactam; RIF, rifampin; STR, streptomycin; SXT, trimethoprim + sulfamethoxazole; TET, tetracycline*.

*R, resistant; S, susceptible; I, intermediate; (-), not tested*.

*MRSA, methicillin-resistant S. aureus*.

*ESBL, extended-spectrum beta-lactamases; KPC, K. pneumoniae carbapenemase; RC, carbapenem-resistant*.

*101, 107, 167, 169, 176,192, 131, 133, and 01 are strain numbers at Laboratory of Basic and Applied Bacteriology-Universidade Estadual de Londrina*.

### Antimicrobial agents

#### Oregano essential oil

OEO was obtained from Ferquima Industry and Commerce of Essential Oils (São Paulo, Brazil). This oil (batch 224) was extracted by steam distillation and its density (0.954 g/mL) and composition (main components: 71% carvacrol, 3% thymol, 4.5% gamma terpinene, 3.5% para-cymene, and 4% beta-caryophyllene) were described in a technical report. A stock solution of 50% OEO was prepared in dimethylsulfoxide (DMSO, Sigma-Aldrich; v/v). DMSO maximum concentration in assays was 1%.

#### Silver nanoparticles

Bio-AgNP were prepared according to a previously established method (Durán et al., 2005, 2006). This method of production has been patented (Patent, 2006, PI 0605681-4A2; http://www.inpi.gov.br). Briefly, bio-AgNP were obtained after reduction of silver nitrate by *F. oxysporum*, strain 551, from the culture collection of the Molecular Genetics Laboratory of ESALQ-USP (Piracicaba, São Paulo, Brazil). *F. oxysporum* was cultivated on media containing 0.5% (w/v) yeast extract (Neogen), 2% (w/v) malt extract (Neogen), 2% (w/v) agar (Neogen) and distilled water at 28°C for 7 days. After growth, the fungal biomass was added to distilled water at 0.1 g/mL and incubated at 28°C for 72 h. Afterwards, the solution components were separated by filtration. AgNO_3_ (Nuclear) at 1 mM was added to fungal-free solution, and the system was incubated for several hours at 28°C in the absence of light. Periodically, aliquots of the solution system were removed and absorptions were measured using an ultraviolet-visible spectrophotometry (Varian Cary 50 Probe); the peak at 440 nm corresponded to the surface plasmon resonance of silver nanoparticles. After bio-AgNP purification, diameter was determined by photon correlation spectroscopy using ZetaSizer NanoZS (Malvern), and zeta potential measurement was performed using the same instrument.

### Antibacterial activity of OEO and Bio-AgNP separately

MIC was determined by the broth dilution method according to Clinical and Laboratory Standards Institute guidelines (CLSI, 2012), with necessary modifications. Tested concentrations of OEO and bio-AgNP ranged from 0.075 to 9.540 mg/mL and 1.91 to 500 μM, respectively. Mueller-Hinton broth (MHB, Difco) alone, MHB plus OEO and MHB plus bio-AgNP were tested as sterility controls, and untreated bacteria inoculated on MHB alone and with 1% DMSO were tested as growth control. After 24 h incubation at 37°C, MIC was defined as the lowest concentration of antimicrobial agent that inhibited visible growth. Minimal bactericidal concentration (MBC) was determined by subculturing 10 μL from the broth dilution MIC, after 24 h of treatment, in Mueller-Hinton agar (MHA, Oxoid) with no antimicrobial agent. MBC was defined as the lowest concentration that kills ≥99.9% of bacteria after 24 h of antimicrobial treatment (NCCLS, 1999). All assays were carried out in triplicate, and at least on three different occasions.

### Antibacterial combination assay

Interaction of OEO and bio-AgNP was determined by broth dilution in double-antimicrobial gradient as described by Traub and Kleber (1975), with modifications. Single colonies of bacterial cultures grown in MHA media were suspended in saline solution (0.9% sodium chloride, w/v, Merck) and adjusted to 0.5 McFarland suspension which corresponds to 1 × 10^8^ colony-forming units/mL (CFU/mL). The inoculum at 1 × 10^8^ CFU/mL was diluted 1:100 in MHB to yield 10^6^ CFU/mL. A volume of 0.05 mL of bacterial inoculum at 10^6^ CFU/mL was added to 0.05 mL of MHB complemented with combination of OEO and bio-AgNP whose concentrations ranged from 0.037 to 0.596 mg/mL and from 7.81 to 125 μM, respectively. Finally bacteria at 5 × 10^5^ CFU/mL were grown in MHB with both antimicrobial agents in combination at 37°C for 24 h. Sterility and growth controls were performed as in the MIC determination assay described above. The interaction of both compounds was analyzed by fractional inhibitory concentrations index (FICI) according Chin et al. (1997), using the following equation: FICI = FIC_OEO_ + FIC_bio-*AgNP*_, where FIC = MIC_combination_/MIC_individual_. FICI is interpreted as “synergistic” when ≤ 0.5, as “additive” when >0.5 and ≤ 1, as “indifferent” (no interaction) when >1 and < 4 and as “antagonist” when ≥4. All assays were carried out in triplicate, at least on three different occasions and against Gram-positive and Gram-negative bacteria, including multidrug-resistant strains.

### Time-kill assay

Time-kill assay was carried out using the viable cells count method, according to NCCLS (1999), with modifications. Three conditions of treatments were tested, bacterial cultures treated with OEO, bio-AgNP and their combination; and a bacterial culture without antimicrobial agent served as growth control. At ten time points (0 h, 30 s, 10 min, 20 min, 30 min, 2 h, 4 h, 7 h, 10 h, and 24 h) of incubation at 37°C, 10 μL from serial dilutions of treated and untreated cultures were transferred to MHA and CFU/mL were determined. All assays were carried out in triplicate, and at least on two different occasions, with three bacterial strains (*S. aureus* ATCC 25923, *E. coli* ATCC 25922 and carbapenemase-producing *E. coli* 131).

### Scanning electron microscopy

*S. aureus* (ATCC 25923, non-MRSA) at 10^9^ CFU/mL were prepared in MHB and added to five tubes (5 mL of bacterial inoculum each). *S. aureus* was exposed to three different treatments; OEO at 0.594 mg/mL was added to the first tube, bio-AgNP at 250 μM were added to the second tube and the two compounds combined were added to the third tube (OEO at 0.297 mg/mL and bio-AgNP at 125 μM, 2x synergistic MIC values to account for the increased cell density used for SEM analyses). Incubation times at 37°C varied for each treatment, 30 min for OEO and 6 h for bio-AgNP and combination of antimicrobials. Two untreated controls were prepared, one with 30 min incubation and another with 6 h. After incubation, 20 μL of each sample were spotted onto poly-L-lysine (Sigma-Aldrich)-coated glass slides. Each slide containing treated or untreated bacteria was fixed (for 20 h) by immersion in 1 mL of 0.1 M sodium cacodylate buffer (pH 7.2) containing 2.5% glutaraldehyde and 2% paraformaldehyde. All samples were then post-fixed in 1% osmium tetroxide for 2 h. All reagents for both chemical fixations were provided from Electron Microscopy Sciences. Post-fixed cells were dehydrated in an ethanol gradient (Sigma-Aldrich) (70, 80, 90 and 100°GL), critical point-dried using CO_2_ (BALTEC CPD 030 Critical Point Dryer), coated with gold (BALTEC SDC 050 Sputter Coater) and observed under a scanning electron microscope (FEI Quanta 200).

### Cytotoxicity assay with human red blood cells (RBC) and HEp-2cells

Hemolytic activity of OEO and bio-AgNP was determined according to Izumi et al. (2012), with necessary modifications. Blood was collected in heparinized tubes (Vacutainer) from a healthy human donor with voluntary consent, which was approved by the human ethics committee (CAAE 47661115.0.0000.5231, No. 1.268.019 – UEL). Erythrocytes were separated by centrifugation (5000 rpm, 4°C, 5 min) and were prepared in phosphate-buffered saline (0.1 M PBS, pH 7.2) at 6% (v/v). PBS was composed of 0.9% (w/v) sodium chloride (Merck), 0.2 M monobasic sodium phosphate (Chemco) and 0.2 M dibasic sodium phosphate (Nuclear). In 96-well plates, 100 μL of RBC at 6% were added to 100 μL of PBS with different concentrations of compounds individually and in combination. After 3 h of incubation at 37°C, supernatants were read at 550 nm to monitor release of hemoglobin. Triton X-100 (Sigma-Aldrich) at 1% was used as control for 100% hemolytic activity, and hemolysis percentage was calculated for each compound concentration.

Cytotoxicity to the human laryngeal epithelial carcinoma cell line HEp-2 was performed by the colorimetric dimethylthiazol diphenyl tetrazolium bromide (MTT, Sigma-Aldrich) assay in 96-well plates, according to the manufacturer's recommendations. HEp-2 cells were grown in RPMI medium 1640 (Gibco) at 37°C in 5% CO_2_ to form a monolayer. Non-adherent cells were removed using PBS and confluent cells were treated with different concentrations of compounds individually and in combination for 24 h at 37°C in 5% CO_2_. After incubation, the medium was removed and each well was washed with PBS. MTT solution (10 μL per well at 1.250 g/mL) was added to all wells, and plates were incubated at 37°C for 2 h. MTT solubilization solution (Sigma-Aldrich) containing 10% Triton X-100 in acidic isopropanol (0.1 N HCl) was added to each well (90 μL per well) to dissolve the dark blue crystals (formazan). After 15 min homogenization, the plate was read at 570 nm. Untreated HEp-2 cells were used as control for 100% viability, and viability percentage was calculated for each compound concentration.

In both cytotoxicity assays, the concentrations of OEO and bio-AgNP ranged 0.037–9.540 mg/mL and 1.95–250 μM, respectively. The 50% cytotoxic concentration (CC_50_) was defined as the antimicrobial concentration required to reduce cell viability by 50% compared to untreated control. CC_50_ of each compound was determined by regression analysis for both cell lines, RBC and HEp-2 tumor cells.

### Statistical method

Results of MIC and time kill assay were analyzed using Wilcoxon or Kruskal-Wallis test followed by Dunn's test. Analyses were performed using R Statistical Software, version 3.1.0 (Foundation for Statistical Computing, Vienna, Austria). Values of *p* < 0.05 were considered significant.

## Results

### Bio-AgNP characterization

Average bio-AgNP size and zeta potential were 77.68 nm and −34.6 mV, respectively (Supplementary Material).

### MIC and antimicrobial interaction

OEO and bio-AgNP inhibited the growth of all bacterial strains tested, including multidrug-resistant strains. The mean MIC for OEO was 0.526 ± 0.130 mg/mL, and the mean MBC was 0.500 ± 0.158 mg/mL, MIC and MBC ranged from 0.298 to 1.193 mg/mL (Table 2). The mean MIC for bio-AgNP was 129.17 ± 55.25 μM ranging from 62.5 to 250μM, and the mean MBC was 154.17 ± 110.46 μM ranging from 62.5 μM to 500 μM (Table 2).

**Table 2:** **Mean of minimal inhibitory and bactericidal concentrations of oregano essential oil and biological silver nanoparticles**.

**Bacteria**	**OEO (mg/mL)**		**bio-AgNP (**μ**M)**	
	**MIC**	**MBC**	**MIC**	**MBC**
*S. aureus* (ATCC 25923)	0.596	0.596	250.0	250.0
*S. mutans* (ATCC 25175)	0.596	0.596	125.0	250.0
*E. coli* (ATCC 25922)	0.596	0.596	62.50	62.50
*S. enterica* Enteritidis (ATCC 13076)	0.298	0.298	62.50	62.50
*K. pneumoniae* (ATCC 10031)	0.596	0.596	62.50	62.50
*K. pneumoniae* (ATCC 700603)	0.596	0.596	125.0	125.0
*S. enterica* Typhimurium UK-1^*^	0.298	0.298	125.0	125.0
MRSA N315	0.596	0.596	250.0	250.0
MRSA 101	1.193	1.193	250.0	500.0
MRSA 107	1.193	1.193	250.0	500.0
*E. coli* ESBL 167	0.596	0.596	125.0	125.0
*E. coli* ESBL 169	0.596	0.596	125.0	125.0
*E. coli* ESBL 176	0.596	0.596	125.0	125.0
*E. coli* ESBL 192	0.596	0.596	125.0	125.0
*E. coli* KPC 131	0.596	0.596	125.0	125.0
*E. coli* KPC 133	0.596	0.596	125.0	125.0
*A. baumannii* CR 01	0.298	0.298	125.0	125.0

*OEO, oregano essential oil; bio-AgNP, biological silver nanoparticles*.

*MIC, minimal inhibitory concentration; MBC, minimal bactericidal concentration*.

*ATCC, American Type Culture Collection*.

* *, ATCC 68169*.

*MRSA, methicillin resistant S. aureus*.

*ESBL, extended spectrum beta-lactamase; KPC, K. pneumoniae carbapenemase*.

CR, Carbapenem-resistant;

*101, 107, 167, 169, 176,192, 131, 133, and 01 are strain numbers at Laboratory of Basic and Applied Bacteriology-Universidade Estadual de Londrina*.

There was no statistical difference between standard and multidrug-resistant strains with regard to MIC values of the two individual compounds (*p* > 0.05). No significant difference (*p* > 0.05) was observed between Gram-positive and Gram-negative sensitivity to OEO, where both bacterial groups were susceptible to this natural compound. Bio-AgNP showed better activity against Gram-negative bacteria (*p* < 0.05), where the mean MIC was 125 ± 56.53 μM in contrast to Gram-positive mean MIC, which was 225 ± 55.90 μM.

In combination, OEO and bio-AgNP showed significantly lower MIC values when compared with individual treatment (*p* < 0.05), where the two compounds together resulted in synergistic or additive antibacterial activity (Table 3).

**Table 3:** **Combinatory effect of oregano essential oil and biological silver nanoparticles and both minimal inhibitory concentrations in combination**.

	**MIC**		**FICI**
**Bacteria**	**OEO (mg/mL)**	**Bio-AgNP (μM)**	
*S. aureus* (ATCC 25923)	0.149	62.50	0.50 (S)
*S. mutans* (ATCC 25175)	0.075	62.50	0.62 (A)
*E. coli* (ATCC 25922)	0.298	15.62	0.75 (A)
*S. enterica* Enteritidis (ATCC 13076)	0.037	31.25	0.62 (A)
*K. pneumoniae* (ATCC 10031)	0.075	15.62	0.37 (S)
*K. pneumoniae* (ATCC 700603)	0.075	62.50	0.62 (A)
*S. enterica* Typhimurium UK-1^*^	0.149	62.50	1.00 (A)
MRSA N315	0.075	125.0	0.62 (A)
MRSA 101	0.596	62.50	0.75 (A)
MRSA 107	0.596	62.50	0.75 (A)
*E. coli* ESBL 167	0.149	15.62	0.37 (S)
*E. coli* ESBL 169	0.149	15.62	0.37 (S)
*E. coli* ESBL 176	0.149	31.25	0.50 (S)
*E. coli* ESBL 192	0.149	31.25	0.50 (S)
*E. coli* KPC 131	0.075	31.25	0.37 (S)
*E. coli* KPC 133	0.075	62.50	0.62 (A)
*A. baumannii* CR 01	0.149	15.62	0.62 (A)

*OEO, oregano essential oil; bio-AgNP, biological silver nanoparticles*.

*ATCC, American Type Culture Collection*.

* *, S. enterica Typhimurium ATCC 68169*.

*MRSA, methicillin resistant S. aureus*.

*ESBL, extended spectrum beta-lactamase; KPC, K. pneumoniae carbapenemase*.

*CR, Carbapenem-resistant*.

*101, 107, 167, 169, 176,192, 131, 133 and 01 are strain numbers at Laboratory of Basic and Applied Bacteriology-Universidade Estadual de Londrina*.

*FICI, fractional inhibitory concentration index*.

*FIC index were interpreted as follows: ≤ 0.5, synergy; > 0.5 to 1.0, addition; > 1.0 to < 4.0, indifference; and ≥ 4, antagonism*.

*(S), Synergistic interaction; (A), Additive interaction*.

### Time-kill curve

**Figure 1:**
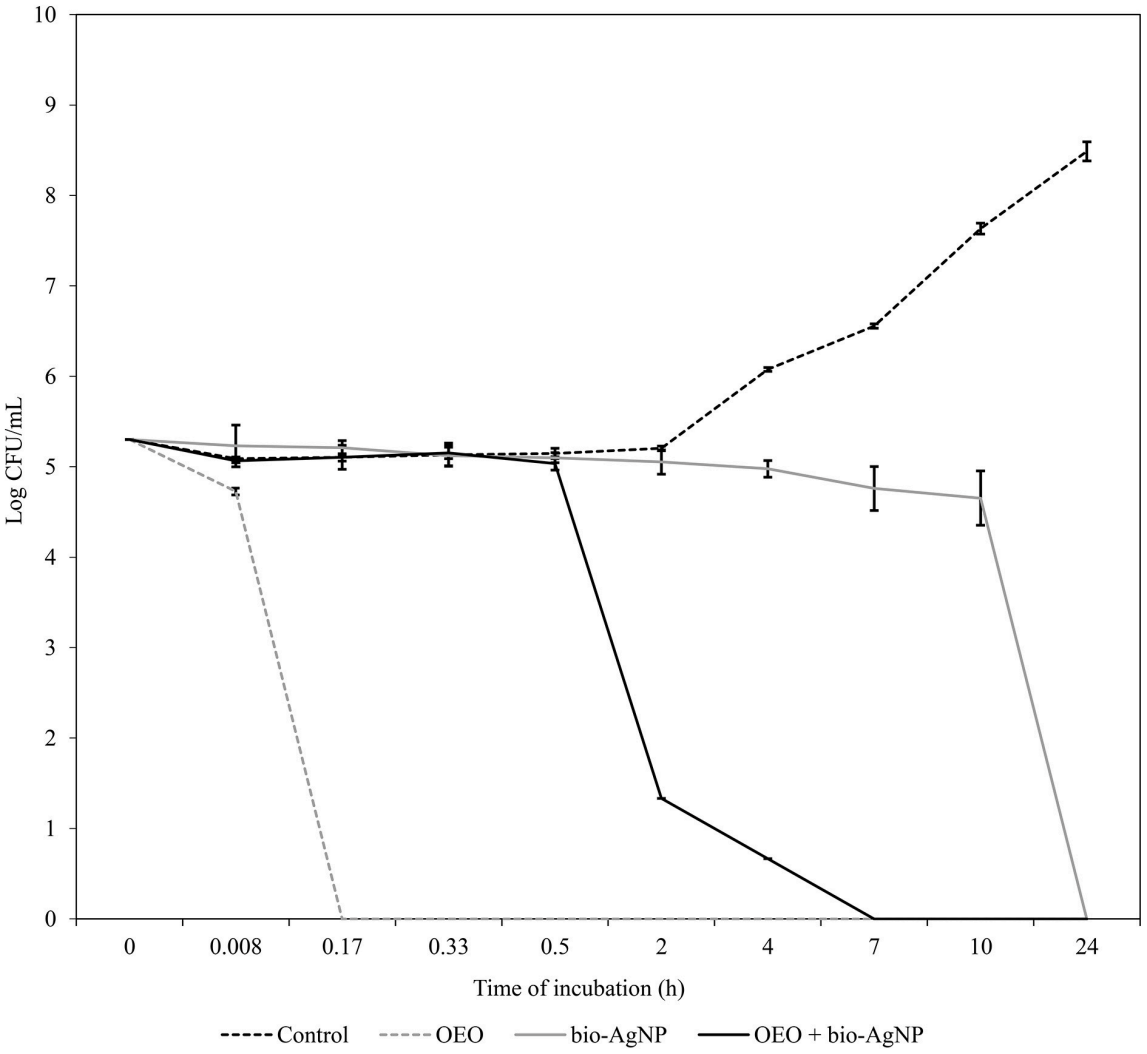
**Time-kill curves of *S. aureus* 25923 (ATCC) exposed to OEO and bio-AgNP individually and in combination at MIC values**. Bacteria at 5 × 10^5^ CFU/mL were exposed to three different treatments; OEO alone (0.596 mg/mL), bio-AgNP alone (250μM), and OEO + bio-AgNP (0.298 mg/mL + 125 μM). Control indicates bacterial growth without antimicrobial compounds. Values of CFU/mL are the mean ± standard deviation.

**Figure 2:**
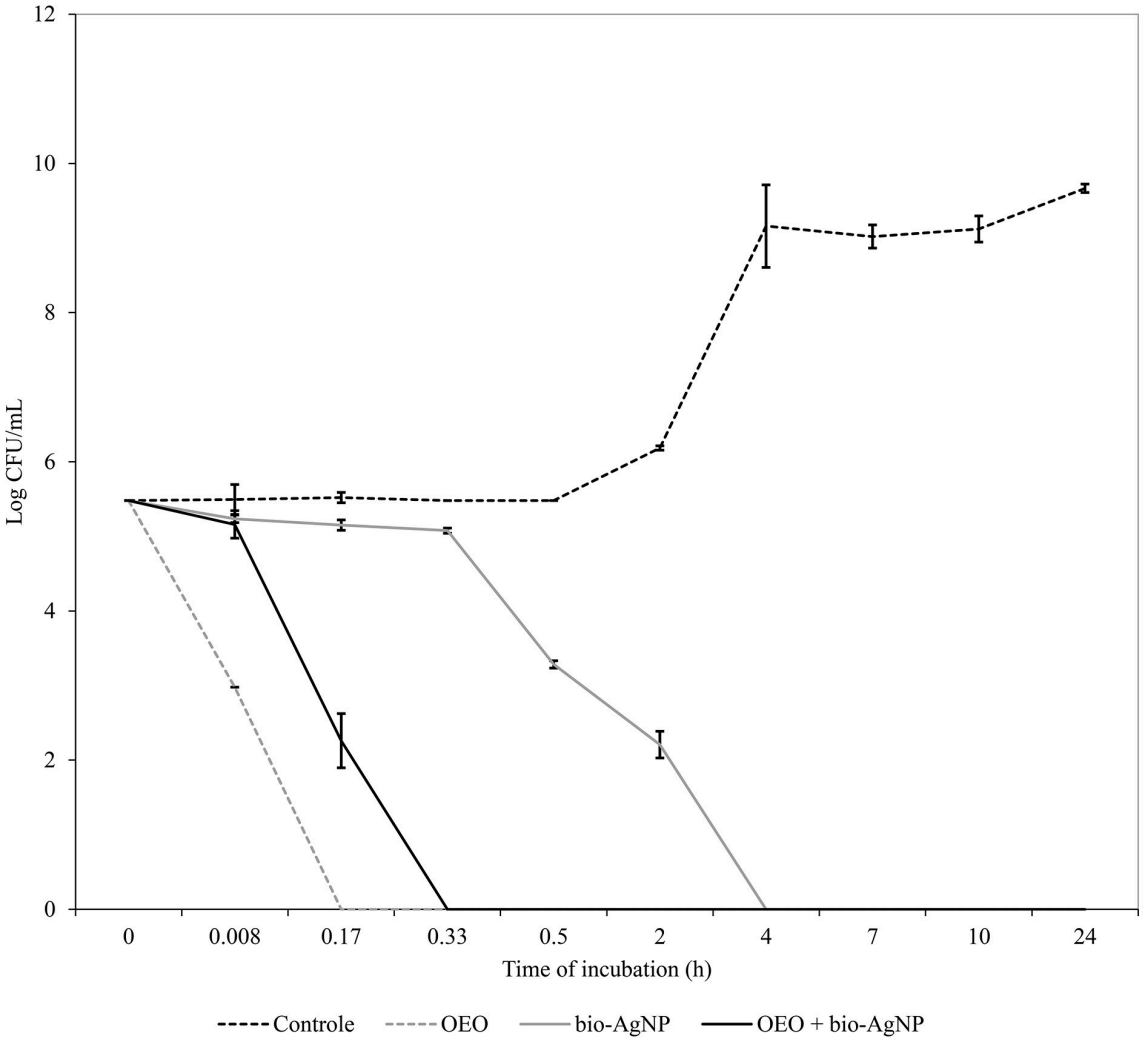
**Time-kill curves of *E. coli* 25922 (ATCC) exposed to OEO and bio-AgNP individually and in combination at MIC values**. Bacteria at 5 × 10^5^ CFU/mL were exposed to three different treatments; OEO alone (0.596 mg/mL), bio-AgNP alone (62.5 μM) and OEO + bio-AgNP (0.298 mg/mL + 15.62 μM). Control indicates bacterial growth without antimicrobial compounds. Values of CFU/mL are the mean ± standard deviation.

**Figure 3:**
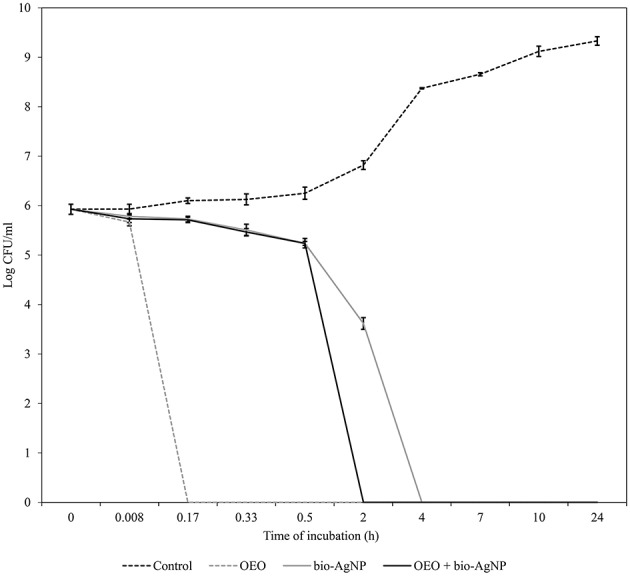
**Time-kill curves of carbapenemase-producing *E. coli* exposed to OEO and bio-AgNP individually and in combination at MIC values**. Bacteria at 5 × 10^5^ CFU/mL were exposed to three different treatments; OEO alone (0.596 mg/mL), bio-AgNP alone (125 μM), and OEO + bio-AgNP (0.075 mg/mL + 31.25 μM). Control indicates bacterial growth without antimicrobial compounds. Values of CFU/mL are the mean ± standard deviation.

OEO reduced the number of CFU/mL rapidly. For all three strains tested (*S. aureus* ATCC 25923, *E. coli* ATCC 25922, KPC-producing *E. coli*), after 10 min of treatment with OEO at MIC (0.596 mg/mL), there were no viable cells (*p* < 0.05; Figures 1–**3**). The bacterial inhibition by OEO was immediate, where after 30 s of treatment, there was a 0.57 log (*p* < 0.05), 1.54 log (*p* < 0.05), and 0.27 log (*p* < 0.05) reduction of *S. aureus* ATCC 25923 (Figure 1), *E. coli* ATCC 25922 (Figure 2), and carbapenemase-producing *E. coli* (Figure 3) cell populations, respectively.

Bio-AgNP at MIC (250 μM) showed bactericidal effect at 24 h to *S. aureus* ATCC 25923 (*p* < 0.05; Figure 1). After 10 h of treatment, the *S. aureus* cell population decreased by 0.57 log (*p* < 0.05); and between 10 and 24 h of treatment, there was a 4.73 log reduction (*p* < 0.05). Gram-negative bacteria lost total viability after 4 h of incubation with bio-AgNP at 62.5 and 125 μM for *E. coli* ATCC 25922 (Figure 2) and carbapenemase-producing *E. coli* (Figure 3), respectively (*p* < 0.05). In both Gram-negative bacteria, after 2 h of exposure to bio-AgNP, there was a significant decrease in CFU/mL, 3.3 and 2.31 log reduction in standard *E. coli* (Figure 2) and KPC-producing *E. coli* (Figure 3), respectively (*p* < 0.05).

The combination of OEO and bio-AgNP led to faster reduction of CFU/mL than in individual treatment with bio-AgNP. Against *S. aureus* ATCC 25923, the combination of the two compounds at synergistic MIC (0.149 mg/mL and 62.5 μM for OEO and bio-AgNP, respectively) showed bacteriostatic activity (data not shown). But the combination with additive MIC values (0.298 mg/mL OEO and 125 μM bio-AgNP) against *S. aureus* ATCC 25923 caused a 3.48 log decrease in cell population in 2 h (*p* < 0.05) and resulted in no viable bacterial cells at 7 h (*p* < 0.05; Figure 1).

Against standard *E. coli* (ATCC 25922), OEO and bio-AgNP at additive MIC (0.298 mg/mL and 15.62 μM, respectively) caused a 2.3 log decrease (*p* < 0.05) within 10 min of treatment and there were no viable cells after 20 min (*p* < 0.05; Figure 2). At 30 min of incubation with synergistic MIC values (0.075 mg/mL and 31.25 μM for OEO and bio-AgNP, respectively), the population of carbapenemase-producing *E. coli* decreased 2.7 log (*p* < 0.05) and after 2 h there was a total reduction of bacterial population (*p* < 0.05; Figure 3).

### Scanning electron microscopy

**Figure 4:**
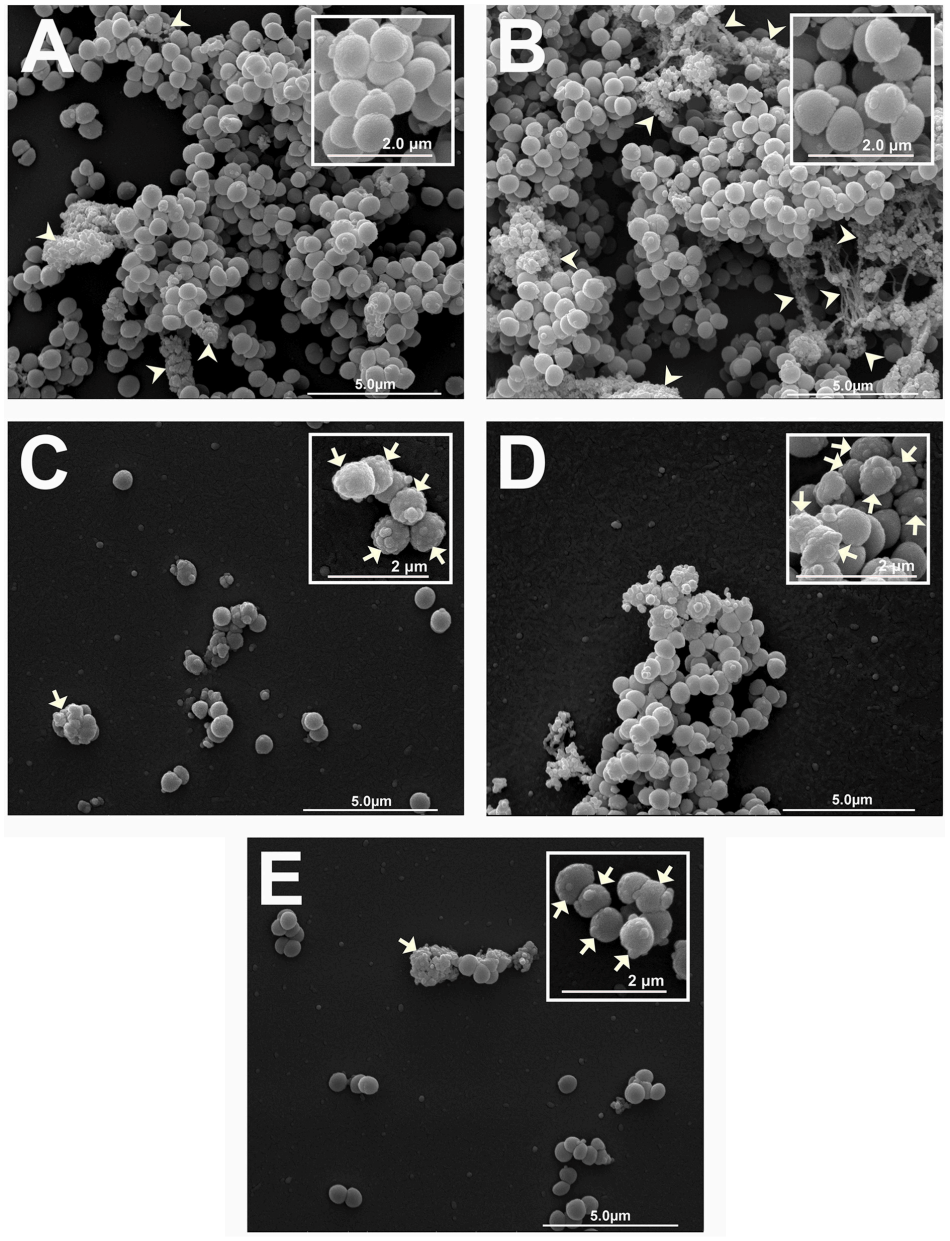
**Scanning electron micrograph of antibacterial effect of OEO and bio-AgNP alone and in combination against *S. aureus* ATCC 25923 (non-MRSA). (A)** Untreated control after 30 min incubation. **(B)** Untreated control after 6 h incubation. **(C)** Treated cells with OEO at 0.596 mg/mL for 30 min. **(D)** Treated cells with bio-AgNP at 250 μM for 6 h. **(E)** Treated cells with combination of OEO and bio-AgNP at 0.298 mg/mL and 125 μM, respectively, for 6 h. Micrographs **(A–E)** show cell density and exopolysaccharide matrix (15,000 x). Inset images show detail of morphological alterations of treated cells and typical cell morphology of untreated controls (30,000 x). Arrows: morphological changes (surface protrusions) and cellular debris. Arrowheads: exopolysaccharide.

To investigate antimicrobial activity against *S. aureus* ATCC 25923 (non-MRSA) by OEO, bio-AgNP and combination of the two treatment, the morphology changes were investigated with SEM. Untreated cells showed their unique shapes (spherical shaped) 30 min (Figure 4A) and 6 h (Figure 4B) after incubation. Inset images showed, seen clearly in the higher magnification, intact surface of *S. aureus* ATCC 25923 (Figures 4A,B - inset). Although cells treated after 30 min with OEO alone was still present, most of them were damaged and extensively disappeared (Figure 4C). Inset showed details, in higher magnification, of morphological changes on the cells surface (Figure 4C - inset). Treatment with bio-AgNP alone after 6 h showed a decrease in cell density and caused morphological changes (Figure 4D). Inset show, in the higher magnification, cells walls covered with substance resulting from serious disruptions in the surface of *S. aureus* ATCC 25923 (Figure 4D - inset). Cells treated with combination of OEO plus bio-AgNP (Figure 4E) showed very deformed cells, with cell debris and damages appeared as cell surface blebbing after 6 h treatment (Figure 4E - inset). Treatments with OEO plus bio-AgNP showed a decrease in cell density, exopolysaccharide, morphology changes, and cell destruction, compared to the non-treated cells. It indicated that this composition have an antimicrobial activity against *S. aureus* by disrupting cells.

### Cytotoxicity assay in human RBC and HEp-2 cells

**Figure 5:**
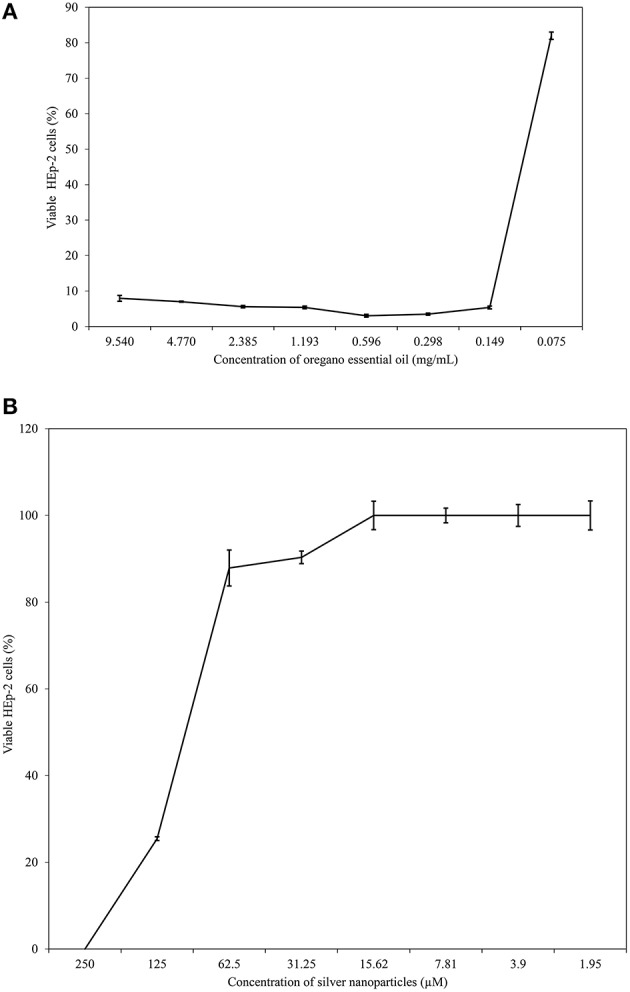
**HEp-2 cell viability at different concentrations of OEO and bio-AgNP individually. (A)** HEp-2 cell exposed to OEO alone ranging from 0.075 to 9.540 mg/mL. **(B)** HEp-2 cell exposed to bio-AgNP alone ranging from 1.95 to 250 μM. Percentage values of cell viability are the mean ± standard deviation.

**Figure 6:**
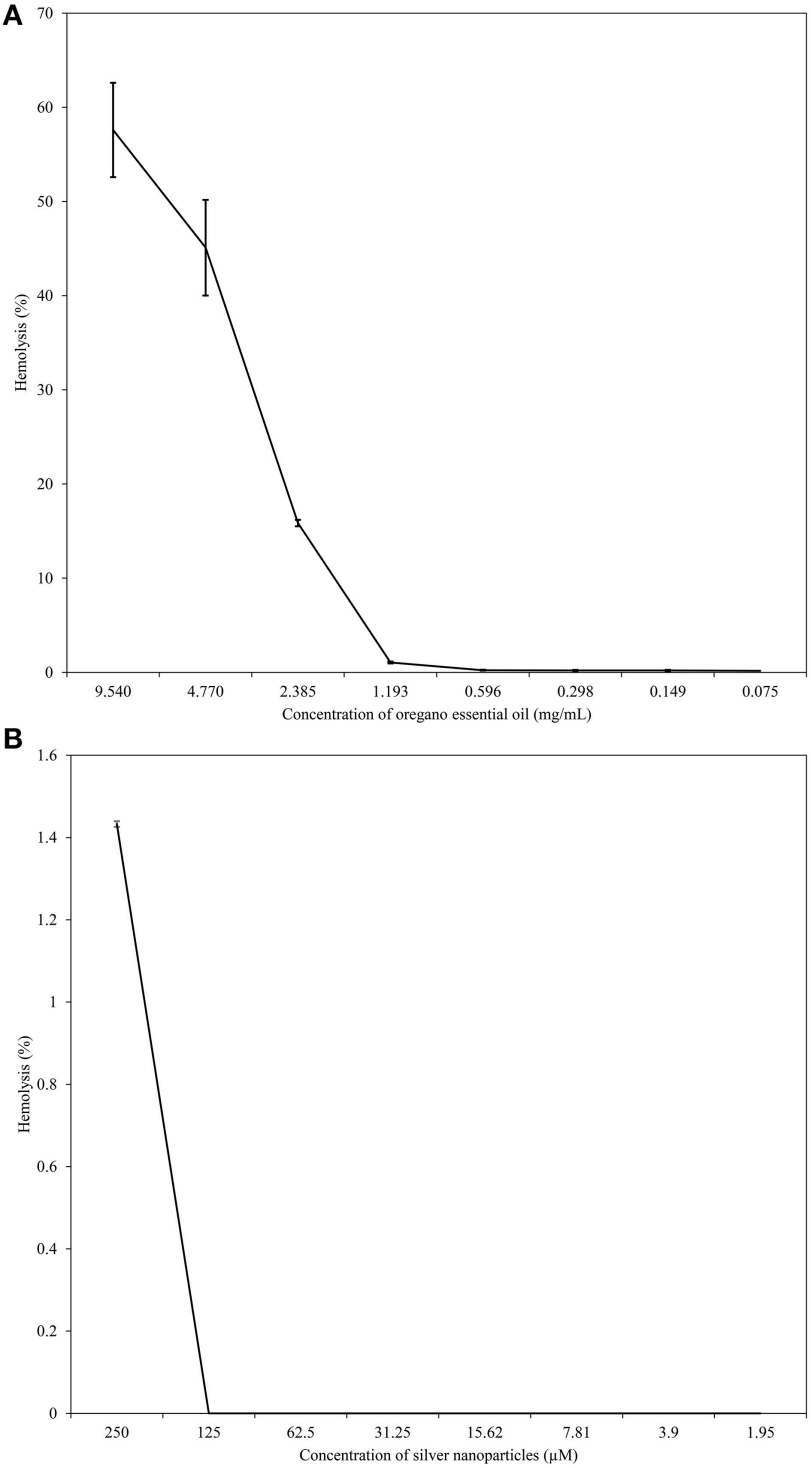
**Hemolytic activity of different concentrations of OEO and bio-AgNP individually. (A)** Human erythrocytes exposed to OEO alone ranging from 0.075 to 9.540 mg/mL. **(B)** Human erythrocytes exposed to bio-AgNP ranging from 1.95 to 250 μM. Values of hemolysis percentage are the mean ± standard deviation.

Results of MTT assay showed that OEO was toxic to tumor cells (HEp-2 cells), reducing cell metabolism by 50% or more at concentrations higher than 0.075 mg/mL (Figure 5A). But this essential oil was less toxic to RBC, and the CC_50_ was 7.519 mg/mL (Figure 6A). CC_50_ of bio-AgNP in HEp-2 cells was 97.22 μM (Figure 5B), and very low hemolytic activity was observed even at the highest concentration tested (250 μM; Figure 6B).

The compounds in combination were non-toxic to HEp-2 cells at concentrations of 0.075 mg/mL and 31.25 μM to OEO and bio-AgNP respectively, or at lower values concentrations. The combination of 0.075 mg/mL of OEO and 125 μM or 62.5 μM of bio-AgNP showed toxicity to HEp-2 cells (Table 4). However, the combination of OEO and bio-AgNP was non-toxic to RBC at all concentrations tested (Table 5).

**Table 4:** **HEp-2 cell viability at different concentrations of oregano essential oil (OEO) and biological silver nanoparticles (bio-AgNP) in combination**.

**Combination**	**Tested concentrations**		**Viable cells (%)**
	**OEO (mg/mL)**	**bio-AgNP (μM)**	
A1	0.596	125.0	0.95 ± 0.69
A2	0.596	62.50	1.36 ± 0.59
A3	0.596	31.25	0.99 ± 0.15
A4	0.596	15.62	1.27 ± 0.40
B1	0.298	125.0	1.18 ± 0.20
B2	0.298	62.50	1.72 ± 0.57
B3	0.298	31.25	1.04 ± 0.20
B4	0.298	15.62	1.99 ± 4.64
C1	0.149	125.0	7.80 ± 2.00
C2	0.149	62.50	21.81 ± 12.19
C3	0.149	31.25	22.94 ± 14.00
C4	0.149	15.62	72.01 ± 17.60^NC^
D1	0.075	125.0	38.68 ± 8.49
D2	0.075	62.50	51.61 ± 3.25
D3	0.075	31.25	94.24 ± 3.67 ^NC^
D4	0.075	15.62	100 ± 0.00^NC^
E1	0.037	125.0	61.54 ± 6.92^NC^
E2	0.037	62.50	82.27 ± 10.55^NC^
E3	0.037	31.25	100 ± 0.00^NC^
E4	0.037	15.62	100 ± 0.00^NC^

*Concentrations of OEO (mg/mL) in combination: A, 0.596; B, 0.298; C, 0.149; D, 0.075; E, 0.037*.

*Concentrations of bio-AgNP (μM) in combination: (1) 125; (2) 62.5; (3) 31.25; (4) 15.62*.

NC *, non-cytotoxic, ±, standard deviation*.

**Table 5:** **Hemolytic activity of oregano essential oil (OEO) and biological silver nanoparticles (bio-AgNP) in combination**.

**Combination**	**Tested concentrations**		**Hemolysis (%)**
	**OEO (mg/mL)**	**bio-AgNP (μM)**	
A1	0.596	125.0	6.49 ± 2.29^NC^
A2	0.596	62.50	2.86 ± 0.11^NC^
A3	0.596	31.25	1.95 ± 0.11^NC^
A4	0.596	15.62	0.49 ± 0.13^NC^
B1	0.298	125.0	7.56 ± 1.16^NC^
B2	0.298	62.50	2.60 ± 1.33^NC^
B3	0.298	31.25	0.77 ± 0.37^NC^
B4	0.298	15.62	0.19 ± 0.32^NC^
C1	0.149	125.0	8.44 ± 0.98^NC^
C2	0.149	62.50	4.58 ± 0.23^NC^
C3	0.149	31.25	0.19 ± 0.17^NC^
C4	0.149	15.62	1.26 ± 0.19^NC^
D1	0.075	125.0	7.75 ± 0.93^NC^
D2	0.075	62.50	2.67 ± 0.73^NC^
D3	0.075	31.25	0.42 ± 0.13^NC^
D4	0.075	15.62	0.00 ± 0.00^NC^
E1	0.037	125.0	5.81 ± 2.27^NC^
E2	0.037	62.50	1.57 ± 0.27^NC^
E3	0.037	31.25	0.42 ± 0.53^NC^
E4	0.037	15.62	0.00 ± 0.00^NC^

*Concentrations of OEO (mg/mL) in combination: A, 0.596; B, 0.298; C, 0.149; D, 0.075; E, 0.037*.

*Concentrations of bio-AgNP (μM) in combination: (1) 125; (2) 62.5; (3) 31.25; (4) 15.62*.

NC *, non-cytotoxic, ±, standard deviation*.

## Discussion

This study showed that OEO has potent bactericidal activity at low concentration with fast action, in agreement with previous studies (Burt and Reinders, 2003; Souza et al., 2010; Betancourt et al., 2012; Alvarez et al., 2014). Busatta et al. (2007) reported for OEO MIC values of 0.23 and 0.46 mg/mL against methicillin-sensitive *S. aureus* and *E. coli* ATCC 25922 respectively. Betancourt et al. (2012) found for OEO a MIC of 0.780 mg/mL against *S. enterica* and 3.125 mg/mL against *E. coli*. OEO MIC values obtained in our study ranged from 0.298 to 1.193 mg/mL (Table 2), which were in line with literature results. Our analysis showed that OEO at a low concentration was bactericidal against all multidrug-resistant bacteria tested, with MBC values of 0.298 mg/mL against *A. baumannii*, 0.596 mg/mL against ESBL and KPC-producing *E. coli* isolates and 1.193 mg/mL against both clinical MRSA isolates.

Time-kill assays showed that OEO reduced cell populations nearly 5 log (after 10 min of treatment) in all three bacterial strains tested (*p* < 0.05), so our results indicated that OEO acted within a few minutes against *S. aureus* ATCC 25923 (Figure 1), *E. coli* ATCC 25922 (Figure 2), and KPC-producing *E. coli* (Figure 3). Burt and Reinders (2003) showed that when *E. coli* O157:H7 at 5 × 10^5^ CFU/mL were exposed to OEO (0.06%, v/v), there were no viable cells after 1 min of treatment. In a study with *S. aureus* isolated from food, Barros et al. (2009) found that OEO at MIC (0.06%, v/v) caused 3 log reduction of initial inoculum (10^8^ CFU/mL) after 2 h of treatment. In our study using an equal initial inoculum (*S. aureus* at 10^8^ CFU/mL) and same OEO concentration (0.596 mg/mL; corresponding to 0.06 %, v/v), there were no viable cells after 30 min (*p* < 0.05), due to a 8 log reduction of *S. aureus* ATCC 25923 population (data not shown).

Some studies have found that terpenoid compounds from OEO are more active against Gram-positive bacteria (Smith-Palmer et al., 1998; Lambert et al., 2001; Stojković et al., 2013). Our results indicated that OEO has broad-spectrum action in agreement with previous studies (Busatta et al., 2007; Rosato et al., 2010; Stojković et al., 2013; Alvarez et al., 2014), where it was equally effective according to MIC (Table 2) and time of action (Figures 1–3) against Gram-positive and Gram-negative bacteria (*p* > 0.05); these results are in line with those obtained by other researchers (Dorman and Deans, 2000; Rosato et al., 2010). This variable susceptibility of bacteria to essential oils can be due to the chemical composition of essential oils, which varies according to seasonal and geographical factors (Medini et al., 2009).

Many studies suggest that essential oils affect the bacterial cell membrane resulting in growth inhibition, since this structure supports the most essential functions in prokaryotes. Bennis et al. (2004) reported cell surface changes such as curling and cracks in *Saccharomyces cerevisiae* treated with thymol. Lambert et al. (2001) suggested that OEO inhibits bacterial growth due to damage to membrane integrity, affecting pH homeostasis and equilibrium of inorganic ions. Suzuki et al. (2015) found physical damage and morphological alterations in *Staphylococcus epidermidis* treated with OEO products. Souza et al. (2010) showed loss of 260 nm-absorbing material and release of potassium ions of *S. aureus* cells treated with OEO, results suggesting increased membrane permeability. In our study, SEM observations confirmed physical damage and considerable morphological alteration as cell surface blebbing in *S. aureus* ATCC 25923 (non-MRSA) cells exposed to OEO for 30 min (Figure 4C), compared to control (untreated bacteria) showing no morphological changes after 30 min (Figure 4A). This analysis by electron microscopy revealed a decrease in cell density of OEO-treated bacteria sample when compared to control sample, according with time kill results at 30 min of incubation (data not shown).

In our RBC toxicity assay, OEO CC_50_ was 7.519 mg/mL (Figure 6A). OEO did not appear to be toxic to RBC, since MIC values ranged from 0.037 mg/mL to 1.193 mg/mL (Tables 2, 3). The lack of cytotoxicity of OEO to erythrocytes has also been found by other investigators. Mancini et al. (2014) reported no hemolytic activity of OEO against bovine erythrocytes, and Cacciatore et al. (2015) showed that carvacrol derivatives did not produce human blood hemolysis at their MIC values. OEO was toxic to HEp-2 cells (Figure 5A), and there are previous studies using tumor cells, including HEp-2 cells, that reported the antitumor activity of OEO or its components (Mehdi et al., 2011; Gautam et al., 2014; Sobral et al., 2014).

Due to its strong antimicrobial property, OEO has potential to replace antibiotics in the food industry and can be incorporated in cosmetic products, among other applications (Alvarez et al., 2014; Suzuki et al., 2015). However, the high volatile character and undesirable organoleptic features can limit its use; therefore, alternatives to solve these limitations are required (Burt, 2004; Alvarez et al., 2014; Hernández-Hernández et al., 2014).

In this study, bio-AgNP exhibited broad-spectrum antibacterial action, inhibiting growth of both Gram-positive and -negative bacteria, in agreement with the literature (Busatta et al., 2007; Jain et al., 2009; Kim et al., 2011). The mean MIC value obtained in our study (129 μM) was according with previous studies using silver nanoparticles produced by F. oxysporum that reported MIC value of 125 μM (Cardozo et al., 2013; Biasi-Garbin et al., 2015). However, we found that Gram-positive bacteria were more tolerant to silver nanoparticles because bio-AgNP MIC values were higher (Table 2) and time of action was slower, compared to Gram-negative bacteria (Figures 1–3), as reported in other studies (Jain et al., 2009; Durán et al., 2010; Agnihotri et al., 2014). Kim et al. (2011) showed that Gram-negative bacteria were more susceptible to silver nanoparticles compared to Gram-positive strains, where there was increased bactericidal activity in time kill assay, loss of protein through the membrane and inactivation of lactate dehydrogenase (LDH), besides marked morphological changes.

The size of the nanoparticles is a key point in their antibacterial activity, where many studies show that antibacterial activity is particle size dependent; antimicrobial activity of nanoparticles increases with smaller size (Panacek et al., 2006; Ayala-Núñez et al., 2009; Agnihotri et al., 2014), and this fact accounts for the variation in MIC data between different studies, limiting comparison.

Our analysis by SEM showed surface protrusions on bio-AgNP treated *S. aureus* cells (non-MRSA) (Figure 4D). Kim et al. (2011) reported that bacterial cells treated with silver nanoparticles had irregular fragments on the surface, a large protein leakage and decrease in bacterial growth; suggesting that fragments can be lost cytoplasmic material resulting in damage to the membrane with a consequent increase in permeability. Studies also report that silver ions have an affinity for thiol groups and may damage respiratory enzymes of cytoplasmic membrane (Holt and Bard, 2005; Li et al., 2010).

In our RBC toxicity assay, bio-AgNP were not cytotoxic to human blood cells (Figure 6B). Our metal compound did not exhibit cytotoxic effect on RBC at the concentrations studied, 250 μM (37.5 μg/mL) to 1.95 μM (0.29 μg/mL), so it was not possible to calculate CC50 for human erythrocytes. Spectrophotometric analysis of supernatants indicated that bio-AgNP did not increase absorbance, demonstrating that little hemoglobin was released from the RBC. Choi et al. (2011) showed that the silver nanoparticles at 700 μg/mL lysed 50% of RBC. According to ASTM E2524-08 (2013)^1^ standards, hemolysis > 5% indicates damage to RBC; in our study, the highest bio-AgNP concentration tested (250 μM) showed only 1.4% hemolysis. Our MTT assay results showed that bio-AgNP was toxic to HEp-2 cells only at high concentrations (CC50 = 97.22 μM), often above that needed to inhibit bacterial growth. Some authors have reported antitumor activity of silver nanoparticles, supporting our finding of bio-AgNP being more toxic to HEp-2 cells than to RBC (Devi and Bhimba, 2012; Antony et al., 2013).

Despite the effective antimicrobial action of bio-AgNP with broad spectrum activity and low cytotoxicity, bacteria can easily develop resistance to these nanoparticles by simple genetic changes (Graves et al., 2015). Thus, alternative studies to work around this problem are needed.

Combined drug treatment is recommended as a strategy to control antimicrobial resistance (Fischbach, 2011; Bollenbach, 2015). Li et al. (2005) observed a synergistic effect of silver nanoparticles combined with amoxicillin against *E. coli*, and other studies have shown a synergistic interaction with other natural alternative compounds such as cinnamaldehyde (Ghosh et al., 2013), eugenol (Biasi-Garbin et al., 2015), and phenazine-1-carboxamide (Cardozo et al., 2013).

Other studies have shown that the OEO acts in synergism with some conventional antimicrobials such as gentamicin against *S. aureus* 29213, *E. coli* 25922, and *A. baumannii* 19606 (Rosato et al., 2010), or has an additive effect in combination with amoxicillin, polymyxin, and lincomycin against ESBL-producing *E. coli* (Si et al., 2008). OEO shows synergistic effects with natural substances such as essential oil of *Thymus vulgare* and *Rosmarinus officinalis* (Stojković et al., 2013; Honório et al., 2015).

In the literature, OEO (Nostro et al., 2004; Si et al., 2008; Amrouni et al., 2014) and silver nanoparticles (Cardozo et al., 2013; Singh et al., 2014; Subashini et al., 2014) were shown to be active against multidrug-resistant bacteria. But this study showed, for the first time, synergistic and additive effects of a combination of OEO and bio-AgNP against bacterial strains, including MRSA and beta-lactamase- or carbapenemase-producing bacteria. Synergistic and additive drug interactions may be potential strategies for controlling resistance evolution, since administration of multiple drugs may disrupt several bacterial functions and thus minimize selection of resistant strains (Yeh et al., 2009; Bollenbach, 2015). In combination, both compounds were effective at con**c**entrations that individually do not affect human erythrocyte (Figure 5), and their synergistic or additive effects neither impact such cells (Table 5). However, OEO combined with bio-AgNP was cytotoxic to HEp-2 (Table 4); as observed for these compounds individually. The combination also showed antibacterial activity in short time (Figures 1–3). Furthermore, the undesirable organoleptic characteristics of OEO are decreased, since the synergistic or additive interaction reduces the concentration of both compounds.

Our results highlight the powerful action of OEO combined with bio-AgNP against Gram-negative bacteria, including those producing ESBL and carbapenemases (Table 3, Figures 2, 3). The combination of the two substances also showed activity against Gram-positive bacteria, since the FICI indicated synergistic and additive interactions (Table 3), and SEM confirmed this antimicrobial action through changes in the *S. aureus* (non-MRSA) cell surface and decrease in cell density (Figure 4E) compared to control (Figure 4B).

In conclusion, the combination of bio-AgNP with OEO resulted in synergistic and additive antimicrobial activities against the multidrug-resistant bacterial strains of *E. coli, A. baumannii* and MRSA. Therefore bio-AgNP combined with OEO has potential to be applied in industry (cosmetics, food and pharmaceutical, for example) and clinical and hospital settings (i.e., for treating wounds and burns infections, or for disinfecting hospital to combat resistant strains as *A. baumannii*).

## Author contributions

SS, conception and drafting of the work, analysis and interpretation of results, writing of this manuscript. LD, conception of the work, analysis of the results (especially with essential oil assays and time-kill curves), review critically of this manuscript. CC, conception and analysis of MTT assay. SY, conception and analysis of MTT assay. CN, design of SEM methodology, image acquisition, analysis and interpretation of SEM results. AD, design of SEM methodology, image acquisition, analysis and interpretation of SEM results. CA, design of SEM methodology, image acquisition, analysis, and interpretation of SEM results. ND, biosynthesis and characterization of silver nanoparticles. GN, analysis and interpretation of results, review critically of this manuscript, final approval of the manuscript version to be published. RK, design of the work, drafting the work, analysis and interpretation of results, guidance in all stages of the work, review critically, and correction of this manuscript, final approval of the manuscript version to be published.

### Conflict of interest statement

The authors declare that the research was conducted in the absence of any commercial or financial relationships that could be construed as a potential conflict of interest.

## Footnotes

^1^ASTM E2524-08 (2013). *Standard Test Method for Analysis of Hemolytic Properties of Nanoparticles*. Available online at: http://www.astm.org/Standards/E2524.htm [Accessed October 17, 2015].

## Abbreviations

OEO: oregano essential. oil
bio-AgNP: biological synthesized silver nanoparticle
RBC: red blood cells.
