# Supplementary material for: Synergistic and Additive Effect of Oregano Essential Oil and Biological Silver Nanoparticles against Multidrug-Resistant Bacterial Strains
Source: Front Microbiol. 2016 May 23;7:760. doi: 10.3389/fmicb.2016.00760 (PMC4876125; doi:10.3389/fmicb.2016.00760)
Supplement: Supplementary file 1 [file DataSheet1.PDF]

Supplementary Material

Synergistic and additive effect of oregano essential oil and biological silver nanoparticles against multidrug-resistant bacterial strains

Sara Scandorieiro, Larissa Ciappina de Camargo, Cesar Armando Contreras, Sueli Fumie Yamada-Ogatta, Celso Vataru Nakamura, Admilton Gonçalves de Oliveira Junior, Célia Guadalupe Tardeli Andrade, Nelson Duran, Gerson Nakazato, Renata Katsuko Takayama Kobayashi*

*** Correspondence:** Renata Katsuko Takayama Kobayashi: kobayashirkt@uel.br

1 Size distribution of bio-AgNP

1.1 Size distribution of bio-AgNP by intensity

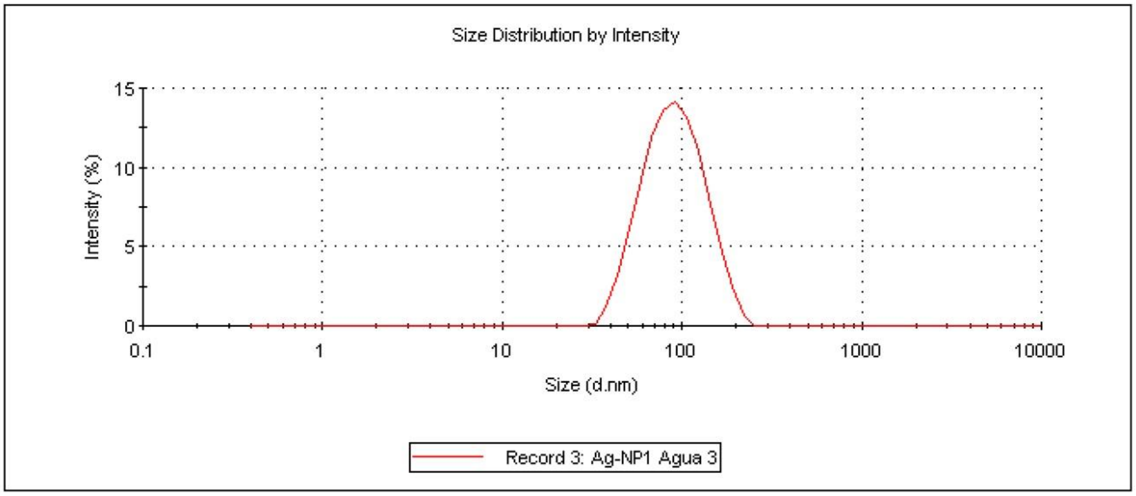

Figure 1. Size distribution by intensity (%) of biologically synthesized silver nanoparticles (bio-AgNP) by *Fusarium oxysporum*. Based on all of the intensities provided by photon correlation spectroscopy, the average diameter of bio-AgNP was 77.68 nm.

1.2 Size distribution of bio-AgNP by volume

Size d.nm	Mean Volume %	Std Dev Volume %	Size d.nm	Mean Volume %	Std Dev Volume %	Size d.nm	Mean Volume %	Std Dev Volume %	Size d.nm	Mean Volume %	Std Dev Volume %
0,4000	0,0		5,615	0,0		78,82	8,4		1106	0,0	
0,4632	0,0		6,503	0,0		91,28	6,2		1281	0,0	
0,5365	0,0		7,531	0,0		105,7	4,3		1484	0,0	
0,6213	0,0		8,721	0,0		122,4	3,0		1718	0,0	
0,7195	0,0		10,10	0,0		141,8	2,0		1990	0,0	
0,8332	0,0		11,70	0,0		164,2	1,3		2305	0,0	
0,9649	0,0		13,54	0,0		190,1	0,8		2669	0,0	
1,117	0,0		15,69	0,0		220,2	0,5		3091	0,0	
1,294	0,0		18,17	0,0		255,0	0,2		3580	0,0	
1,499	0,0		21,04	0,0		295,3	0,1		4145	0,0	
1,736	0,0		24,36	0,4		342,0	0,0		4801	0,0	
2,010	0,0		28,21	2,4		396,1	0,0		5560	0,0	
2,328	0,0		32,67	6,7		458,7	0,0		6439	0,0	
2,696	0,0		37,84	11,2		531,2	0,0		7456	0,0	
3,122	0,0		43,82	14,0		615,1	0,0		8635	0,0	
3,615	0,0		50,75	14,4		712,4	0,0		1,000e4	0,0	
4,187	0,0		58,77	13,1		825,0	0,0				
4,849	0,0		68,06	10,9		955,4	0,0				

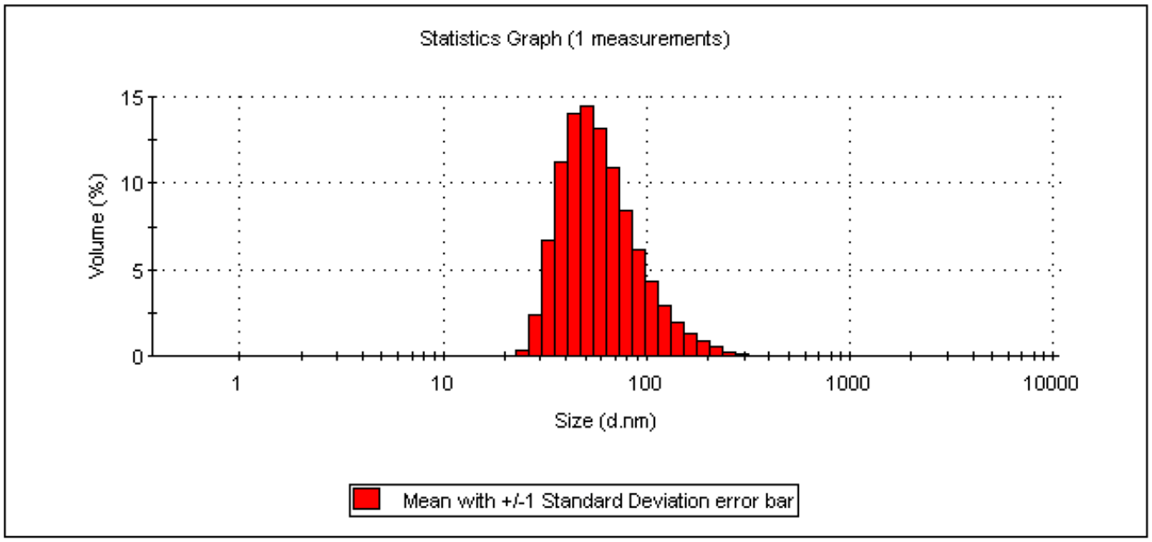

Figure 2. Size distribution by volume (%) of biologically synthesized silver nanoparticles (bio-AgNP) by *Fusarium oxysporum*. Both graph and table describe the size distribution based on the total solution composition.

2 Transmission electron micrograph of bio-AgNP

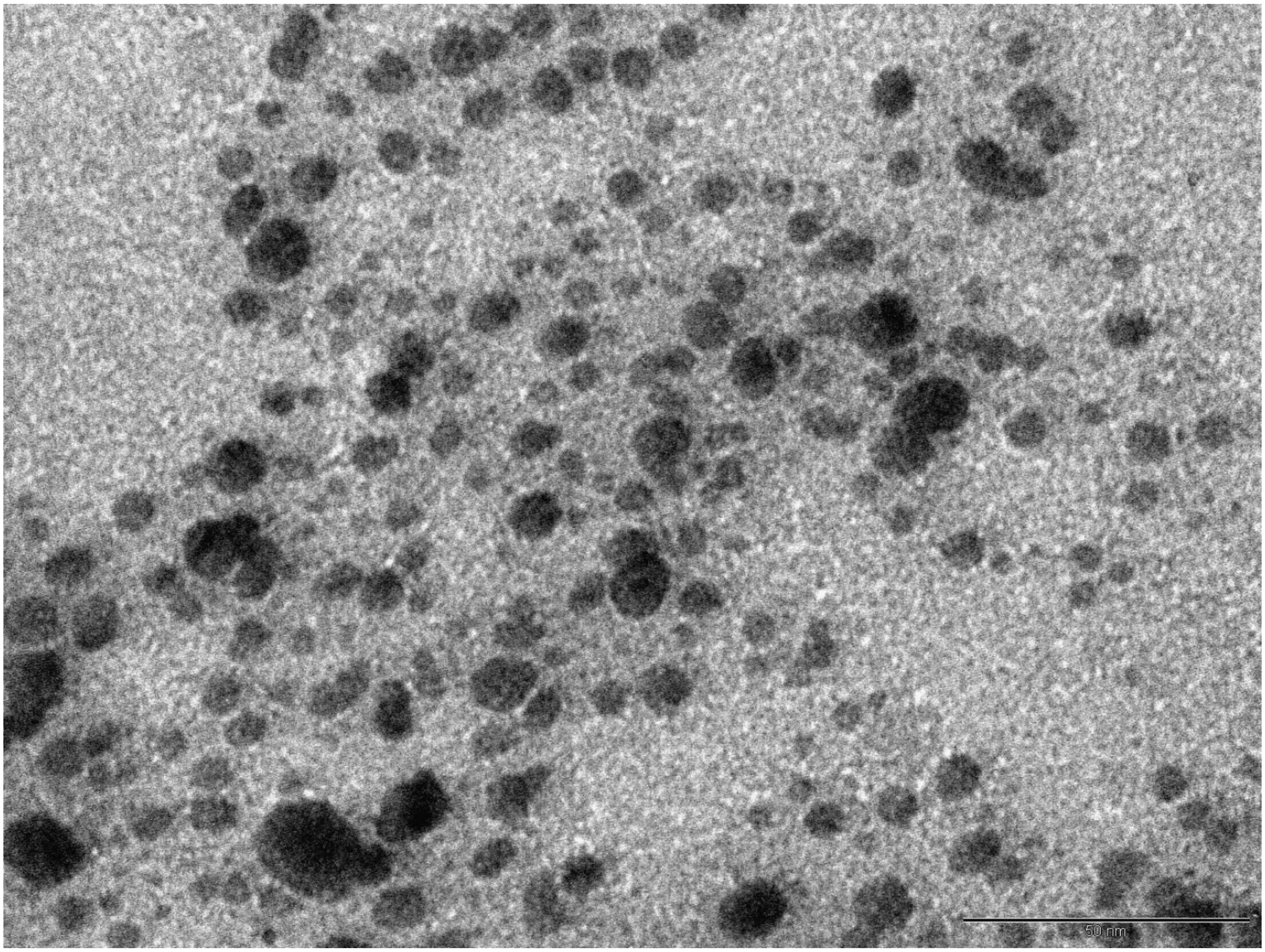

Figure 3. Transmission electron micrograph of biological silver nanoparticles (bio-AgNP) produced using *Fusarium oxysporum*. In this micrograph, spherical nanoparticles were observed.

3 Zeta potential distribution of bio-AgNP

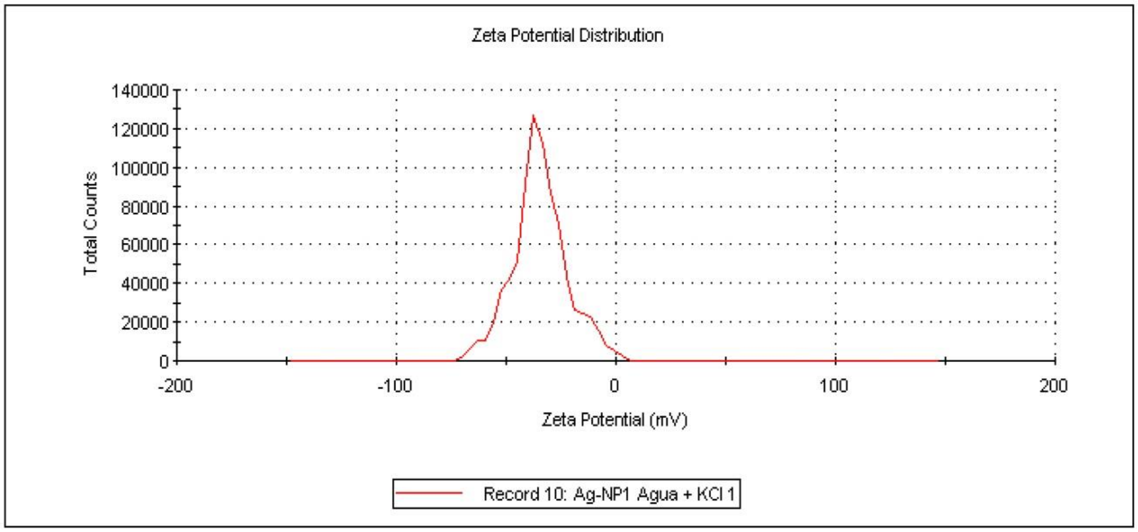

Figure 4. Zeta potential distribution of biological silver nanoparticles (bio-AgNP) determined using Zetasizer NanoZS (Malvern). The average zeta potential value of bio-AgNP was -34.6 mV indicating good stability of nanoparticles.
